# Cryptococcus Meningoencephalitis With Severe Localizing Neurological Deficits Mimicking a Large Right Middle Cerebral Artery Infarct in a HIV Patient

**DOI:** 10.7759/cureus.14781

**Published:** 2021-04-30

**Authors:** Fadi Tahhan, Argin Haritounian, Lisa Duong, Jessica Haugen, Antonio K Liu

**Affiliations:** 1 Internal Medicine, Adventist Health White Memorial, Los Angeles, USA; 2 Neurology, Adventist Health White Memorial, Los Angeles, USA; 3 Family Medicine, Adventist Health White Memorial, Los Angeles, USA

**Keywords:** cryptococcus meningoencephalitis, mimicking infarct, hiv

## Abstract

Immunocompromised patients with Cryptococcal meningoencephalitis can develop focal neurological signs and symptoms. Stroke and abscess are usually the leading etiologies. Definitively localized non-fluctuating deficits mimicking a large middle cerebral artery (MCA) infarct without corresponding MRI findings is rare. Localized lobar cerebritis may be the underlying etiology. Despite having many different kinds of sequences, a significant pathological process can still evade MRI’s detection. Diffusion-weighted imaging (DWI) abnormality has also been seen in pathology other than ischemic stroke.

## Introduction

Cryptococcus meningoencephalitis is a common occurrence among immunocompromised individuals. Stroke-like presentation has been documented as an adverse effect of cryptococcus [1,2]. Stroke mimic, however, is rare. Diffusion-weighted imaging (DWI) is sequence of choice to detect acute ischemic stroke. It visualizes cytotoxic edema associated with an ischemic process. DWI can be positive in other pathologies as well. In fungal infection, DWI abnormality can be seen in cerebritis that eventually developed into an abscess [3,4]. For a disease process to evade MRI’s detection, its pathophysiology could be non-ferromagnetic in nature. A functional/metabolic suppression without actual infarct or hypoperfusion may have a normal MRI. Such phenomena can be seen in postictal state, cortical spreading depression, lupus cerebritis among others. We present an immunocompromised male with cryptococcal meningoencephalitis who developed signs and symptoms of a large right middle cerebral artery (MCA) ischemic infarct without the corresponding radiological finding of a large stroke. Instead of a large DWI deficit in the right MCA territory, only a 5 mm DWI abnormality was identified. The patient subsequently improved with treatment over time.

## Case presentation

**Figure 1:**
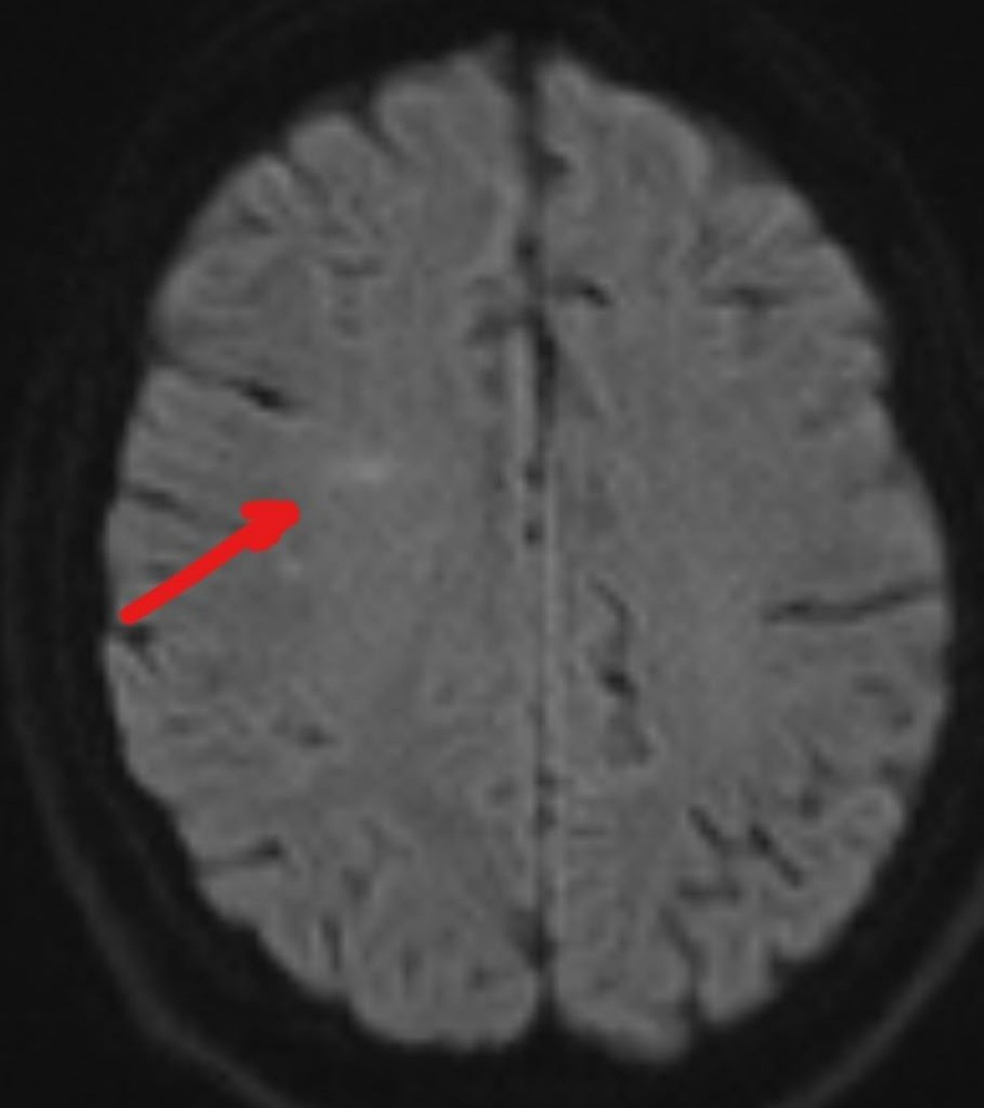
DWI of head MRI, Day 4. DWI: diffusion-weighted imaging.

A 27-year-old male presented to the Emergency Department with a headache and intermittent altered mental status for the past two weeks. He was noted to be oriented and coherent with non-focal examination. Initially, past medical history was scarcely provided. He had a low-grade fever and CT head without contrast upon arrival showed no acute finding. He had an unremarkable complete blood count, comprehensive metabolic panel and negative toxicology study. His initial white cell count was 9.5 K/uL. He tested positive for HIV with a CD4 count of 185 and his serum culture showed yeast with a Cryptococcus titer positive at 1:160. He was started on IV fluconazole. On Day 4 of admission, he suffered a seizure and a neurological consultation was obtained. At that moment, he was postictal, lethargic, but remained oriented to name, place, and situation. His vital signs were stable with no fever. His neck was stiff and he experienced a moderate headache with photophobia. He had a normal and non-focal cranial nerve and motor system examination. Intravenous levetiracetam 750 mg twice daily was initiated. The lumbar puncture (LP) showed an opening pressure greater than 55 cm H2O. A cerebral spinal fluid (CSF) analysis showed 3 WBC/mm^3^, 2 RBC/mm^3^, 43 mg/dL protein and 32 mg/dL glucose (serum glucose was 117 mg/dL). The CSF Cryptococcus titer was positive at a 1:160 titer. Studies for toxoplasmosis, coccidioidomycosis, CMV and JC virus were negative. His headache improved after the spinal tap. EEG at that moment only showed diffuse slowing. An MRI of the head without contrast showed a faint DWI signal abnormality in the right frontal subcortical area (Figure 1), and all other sequences were non-significant.

**Figure 2:**
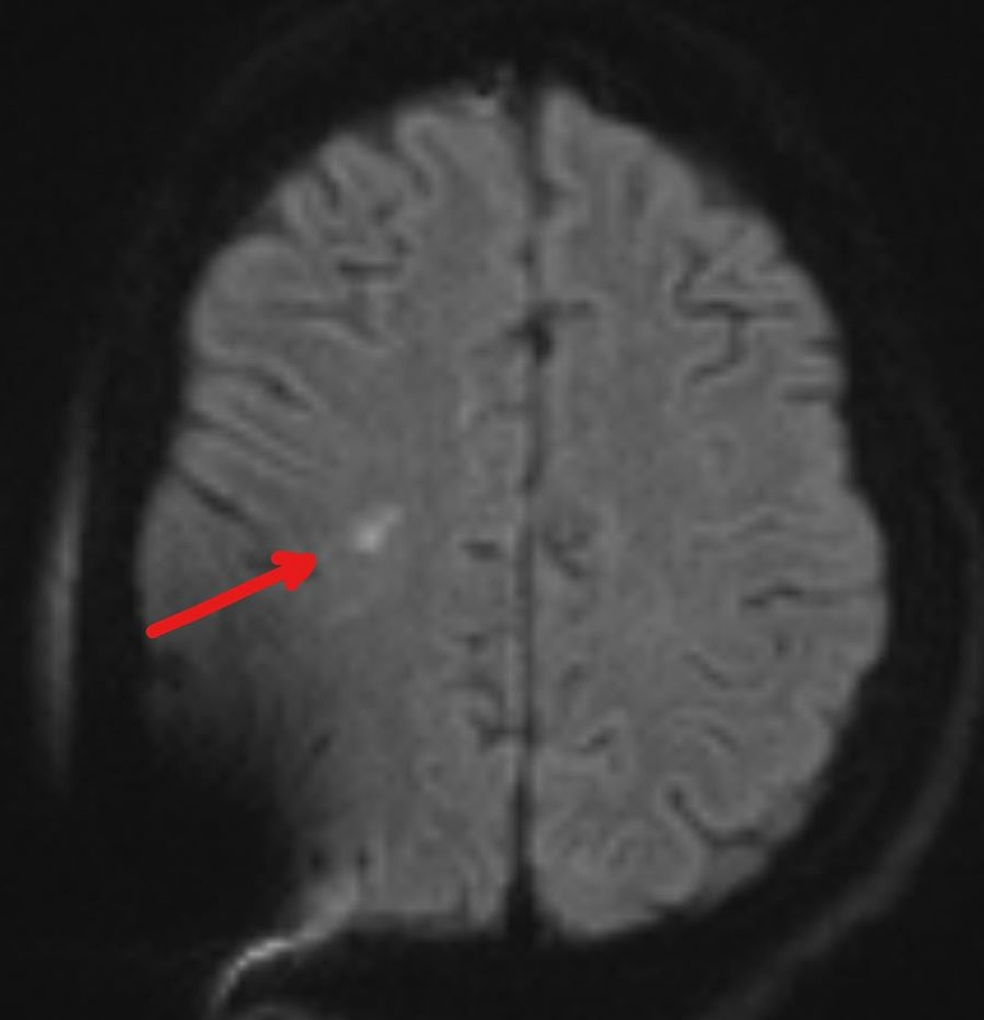
DWI of head MRI, Day 6. DWI: diffusion-weighted imaging.

**Figure 3:**
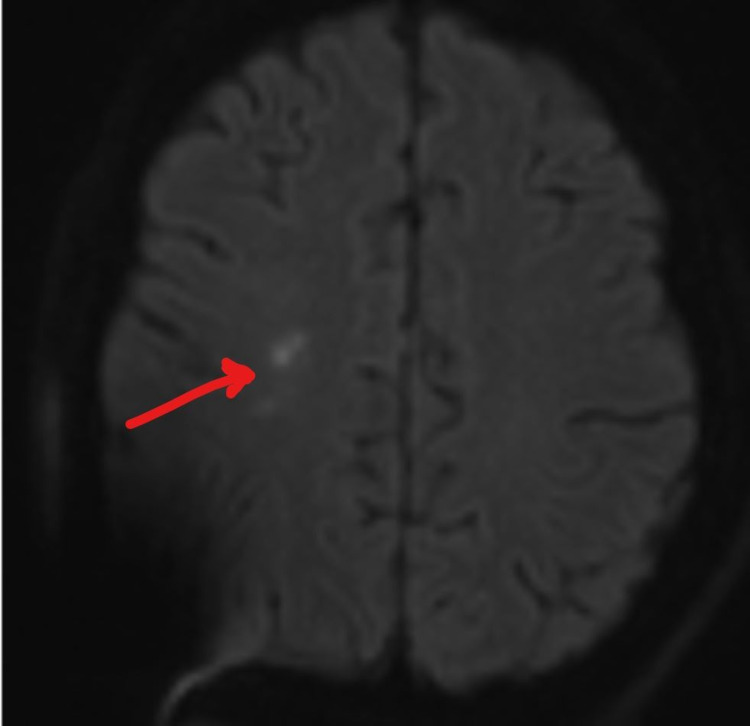
DWI of head MRI, Day 10. DWI: diffusion-weighted imaging.

**Figure 4:**
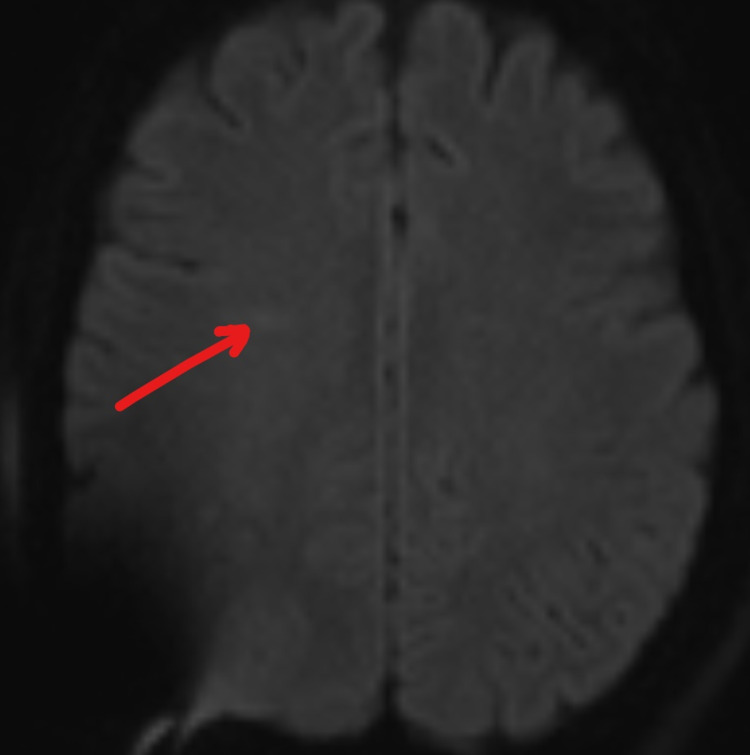
DWI of head MRI, Day 27. DWI: diffusion-weighted imaging.

On Day 6, he experienced rapid deterioration requiring intubation. His repeat LP still showed opening pressure greater than 55 cm H2O and an external ventricular drain (EVD) was placed. He began to develop left upper extremity weakness; motor strength was 4/5 and there was no neglect nor gaze preference initially. On a repeat MRI of the head (Figure 2), the original small DWI lesion has resolved. Instead, a new small DWI signal abnormality in the right frontal deep white matter area posterior to the previous abnormality was identified. His symptoms progressed the next few days. By Day 10, his National Institute of Health Stroke Score (NIHSS) was 20. The patient displayed a dense left hemiparesis, a complete left neglect, a left visual field deficit and rightward gaze preference; signs and symptoms normally found only in a large right MCA territory ischemic stroke. A repeat MRI (Figure 3) showed no extension of infarct. CT angiogram was negative for large vessel occlusion, stenosis or vasculitis. Sonographic studies revealed no vasospasm. His symptoms slowly improved, reaching an NIHSS zero by Day 22 of hospitalization. An MRI performed on Day 27 showed resolution of the lesion and the patient was discharged home (Figure 4). His infection has been managed by an infectious disease specialist with intravenous amphotericin B and HAART treatment. EEG performed serially only showed generalized slowing; there was never any focal slowing. 

A telemedicine consultation was conducted three months post-discharge and the patient was reportedly asymptomatic and partially returned to previous level of activity and work. Lowered endurance was his only complaint. He reported compliance to medical care and medications.

## Discussion

Cerebral vascular accident is known to be a sequela of cryptococcal meningoencephalitis. However, stroke mimic presentations are rare. The patient developed signs and symptoms that resembled a large right MCA stroke a few days after admission. MRI revealed a very small area of abnormal DWI which was inconsistent with the profound degree of neurological deficits. Despite having a seizure episode, EEG did not capture any asymmetry. Imaging studies done with contrast and sonography were negative for vasculitis or vasospasm. The only pathology we were able to identify was an overwhelming fungal meningoencephalitis in a newly diagnosed immunocompromised patient. With no irreversible catastrophic lesion identified, the leading diagnosis was a dysfunctional right cerebral hemisphere due to encephalitis/cerebritis. The patient recovered over time with aggressive antifungal and supportive treatment.

Stroke mimic is a phenomenon when physical examination findings localized to a certain cerebral artery distribution contrary to negative objective studies. This patient has several unique features. First, his findings are suggestive of a very large right MCA stroke, yet objective studies only yield a very small DWI abnormality. Lesion of such size would have been rather asymptomatic; there is a mismatch. Secondly, given the severity of neurological deficits, no objective studies had been able to provide a pathology of such level. Last but not least, assuming cerebritis was the underlying pathology, the fact that these deficits localized only to the right MCA territory is puzzling.

No report has documented a true stroke mimic. Current literature reporting similar clinical cases all have larger and more malignant lesions and strokes than our patient. The patient described by Oberman [5] had radiological findings of vasculitis and large bilateral cerebellar strokes. The patient described by Deming [6] has hydrocephalus and CVA was noted at the splenium of the corpus callosum. Zhou reported an elderly HIV negative patient with cryptococcal meningitis “imitating” a cerebral infarct. However, MRI revealed extensive bilateral ischemic changes [7]. Saul described a healthy young man with cryptococcal meningitis and hemiparesis. Head CT was negative but MRI was not done [8]. Wilson reported a young immunocompetent female with disseminated cryptococcosis and right hemiparesis with MRI confirming the presence of multiple lesions in the left hemisphere [9]. No report has reported a major examination/MRI finding mismatch.

An alternative explanation for such mismatch may be due to the fact that DWI findings have been reported in pathology besides ischemic stroke. DWI abnormality has been documented in patients with fungal cerebritis which was a precursor to abscess formation [3,4,10]. However, these findings were rather extensive and non-subtle in their cases. It is possible our patient was having cerebritis that would eventually lead to an abscess formation in the right hemisphere within the area covered by MCA. Aggressive antifungal treatment may have halted the abscess formation, which may have explained the subtlety of DWI signal as well as the localization to the right MCA territory.

From a metabolic standpoint, suppressed neuronal function may also be another alternative explanation. Conditions like postictal state, migraine cortical spreading depression, and lupus cerebritis are known to have relatively diminutive to no MRI findings. In these situations, a functional MRI or fluorodeoxyglucose PET may be revealing [11]. Such studies were not available to us and are usually outpatient-based. EEG is known to be a powerful tool to detect hypometabolic state, but it was negative serially for our patient, demonstrating no focal hypometabolism.

## Conclusions

In conclusion, this patient has a large right MCA territory stroke mimic. The few reports available all have more subjective findings than our patient. In general, although focal neurological finding is associated with stroke, abscess or other demonstrable lesions, a major mismatch between physical examination findings and objective result can exist. Aggressive treatment remains the primary directive while further investigations take place.
